# Supplementary figures and images for: ER-to-lysosome-associated degradation acts as failsafe mechanism upon ERAD dysfunction
Source: EMBO Rep. 2024 May 21;25(6):2773–85. doi: 10.1038/s44319-024-00165-y (PMC11169228; doi:10.1038/s44319-024-00165-y)

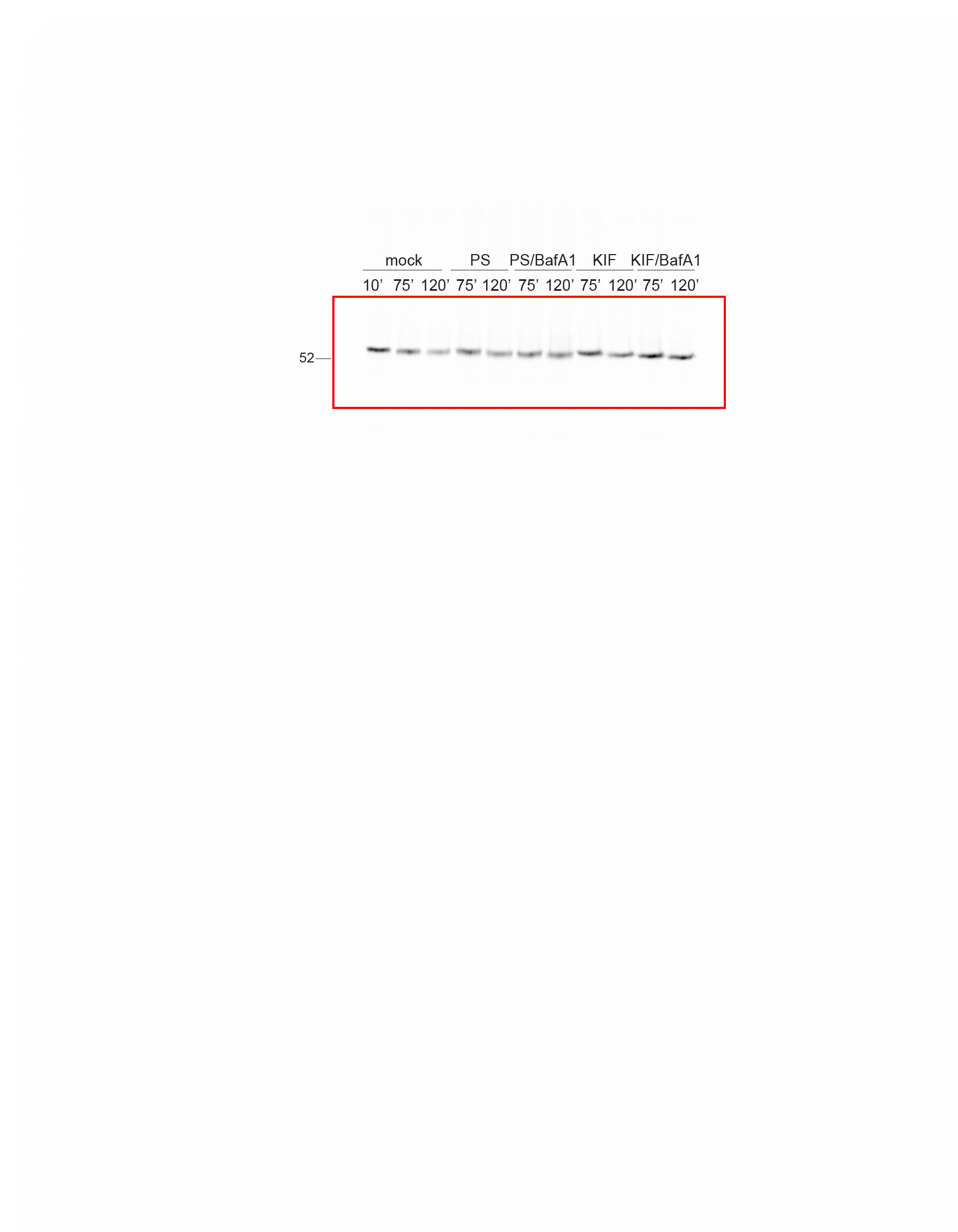

Supplement: Supplementary file 1 — Source data Fig. 2 [file 44319_2024_165_MOESM1_ESM.zip › Figure 2/2A/gel 35S.tif]

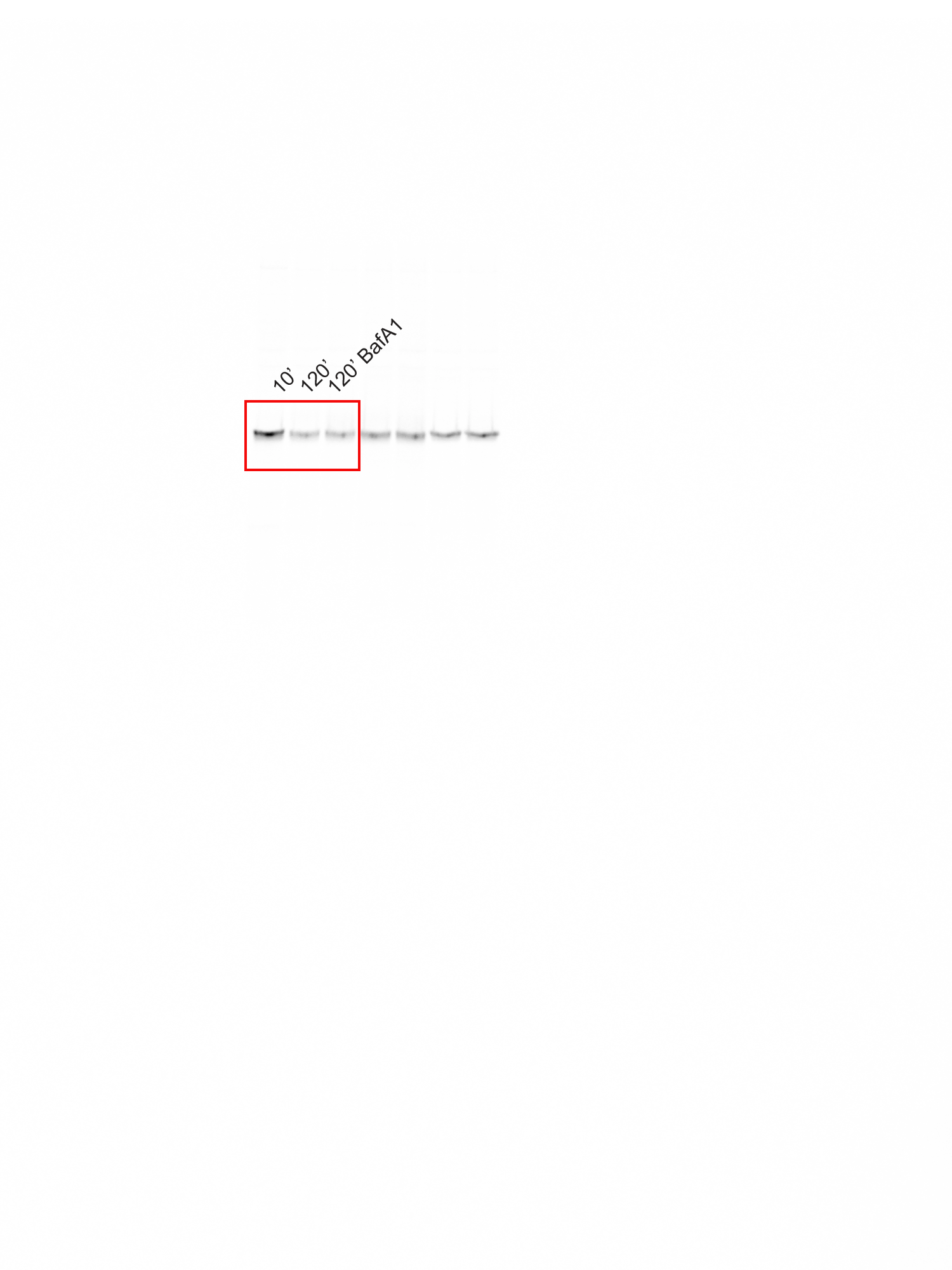

Supplement: Supplementary file 1 — Source data Fig. 2 [file 44319_2024_165_MOESM1_ESM.zip › Figure 2/2D/gel 35S.tif]

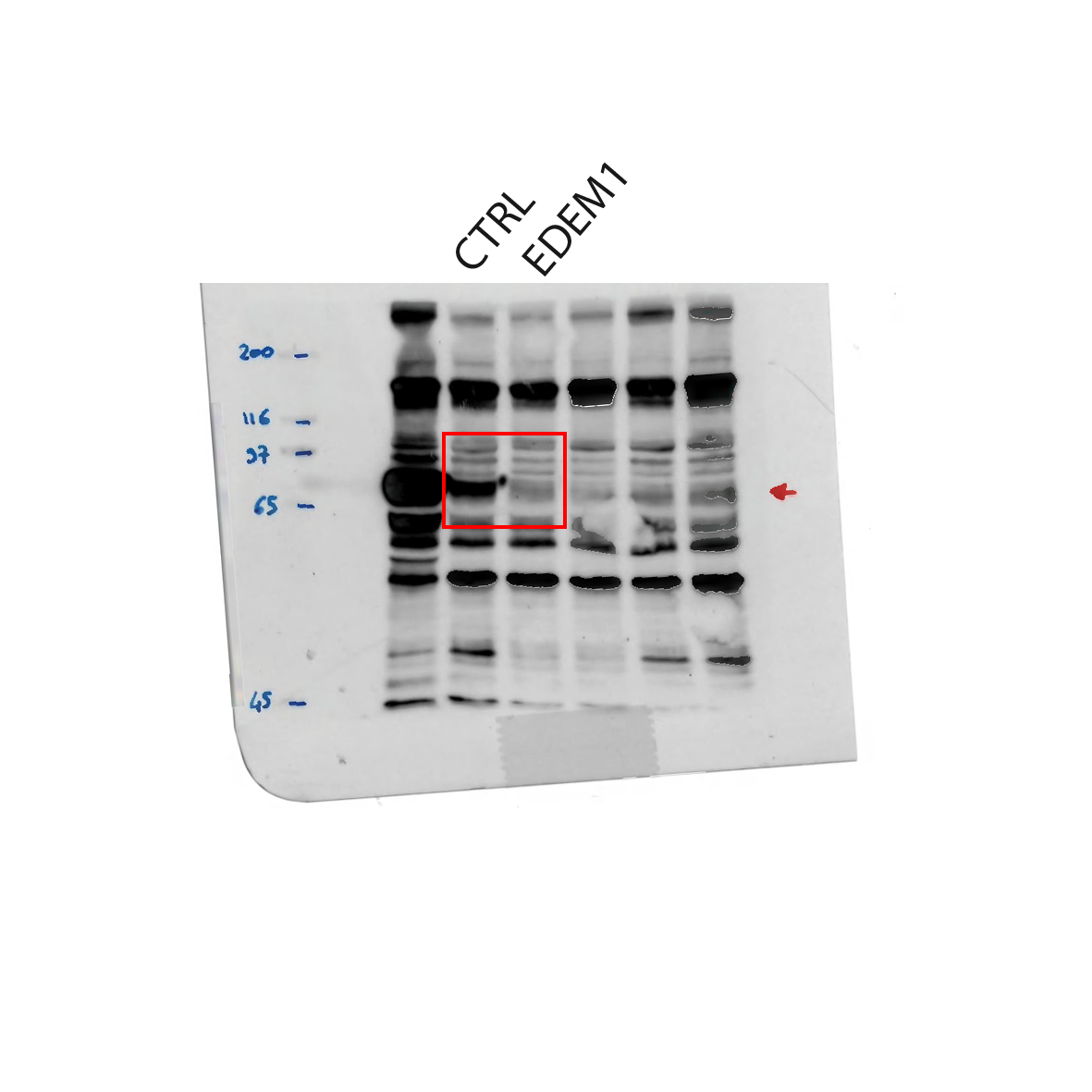

Supplement: Supplementary file 2 — Source data Fig. 3 [file 44319_2024_165_MOESM2_ESM.zip › Figure 3/3A/WB.tif]

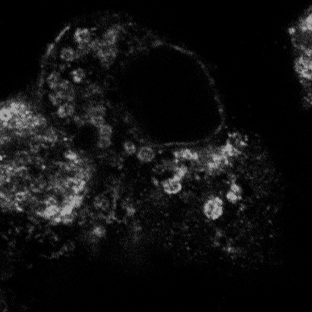

Supplement: Supplementary file 2 — Source data Fig. 3 [file 44319_2024_165_MOESM2_ESM.zip › Figure 3/3B/sh edem ha.tif]

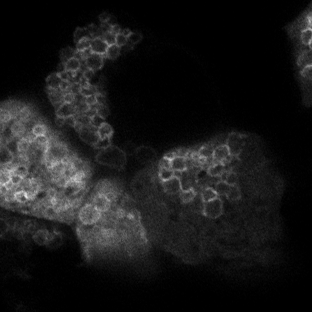

Supplement: Supplementary file 2 — Source data Fig. 3 [file 44319_2024_165_MOESM2_ESM.zip › Figure 3/3B/sh edem lamp.tif]

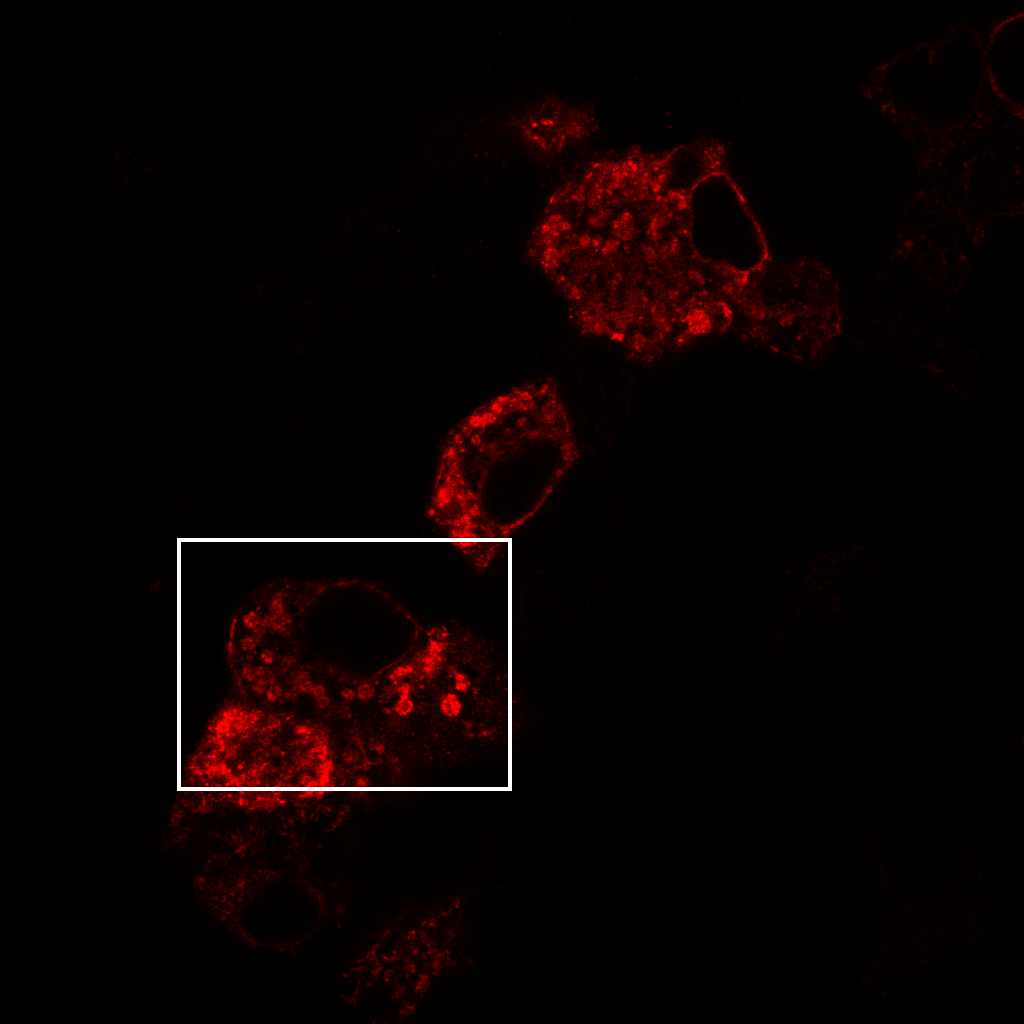

Supplement: Supplementary file 2 — Source data Fig. 3 [file 44319_2024_165_MOESM2_ESM.zip › Figure 3/3B/shEDEM ha.tif]

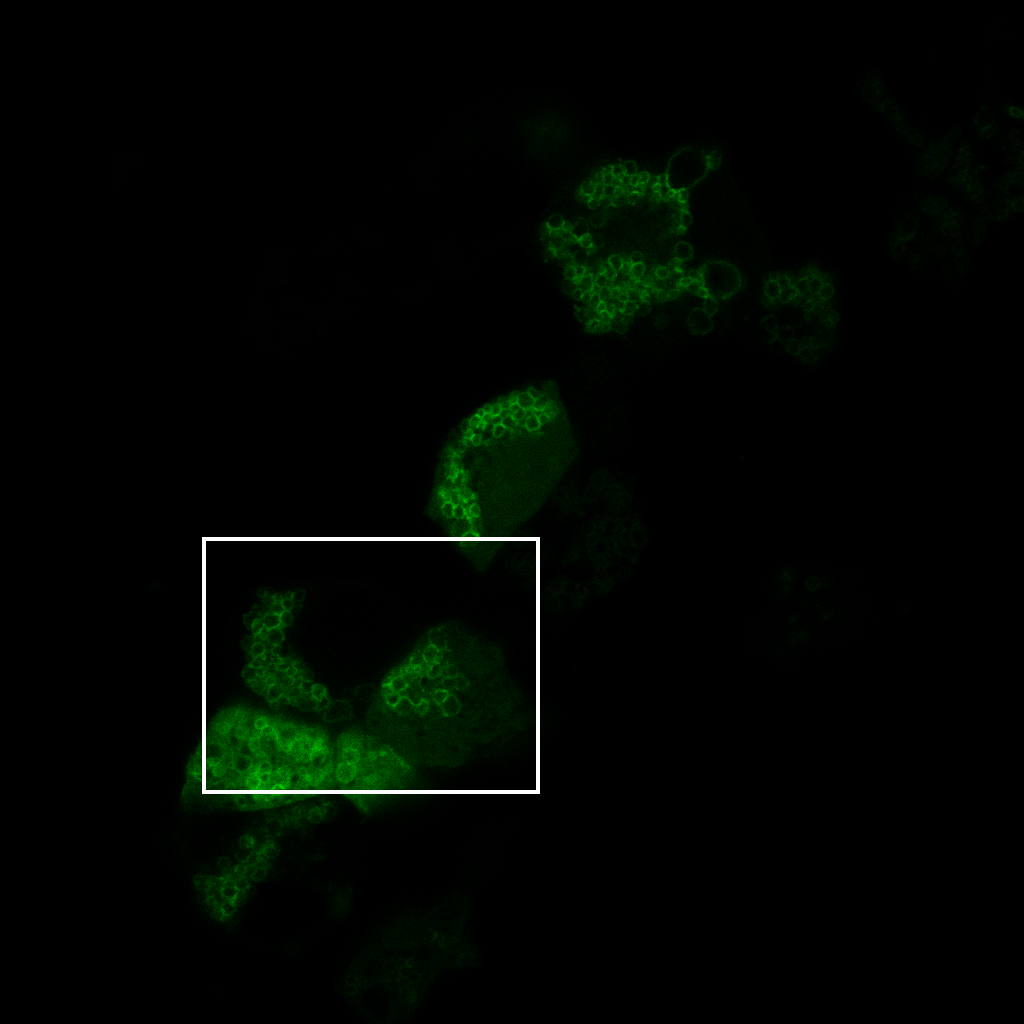

Supplement: Supplementary file 2 — Source data Fig. 3 [file 44319_2024_165_MOESM2_ESM.zip › Figure 3/3B/shEDEM lamp.tif]

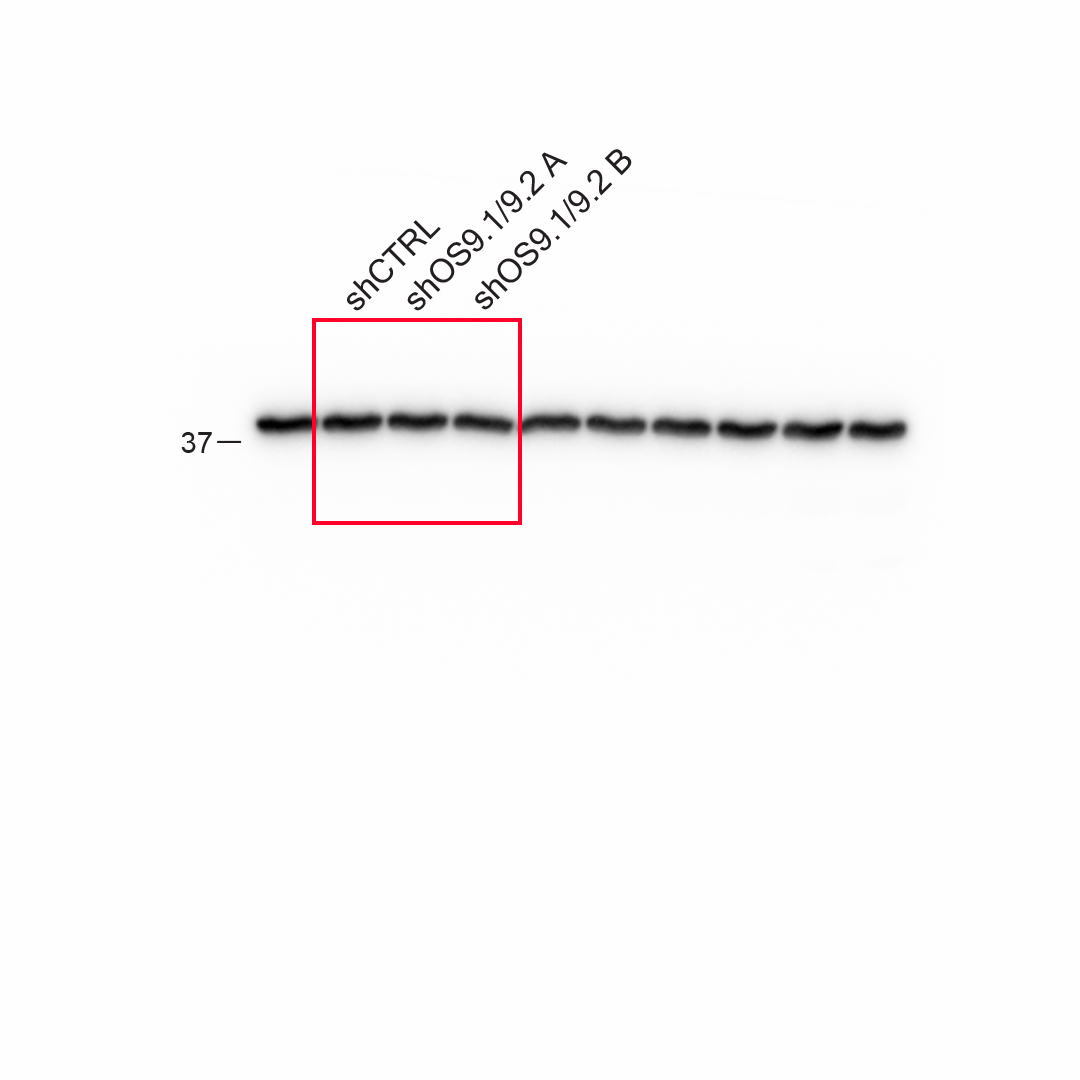

Supplement: Supplementary file 2 — Source data Fig. 3 [file 44319_2024_165_MOESM2_ESM.zip › Figure 3/3D/WB GAPDH.tif]

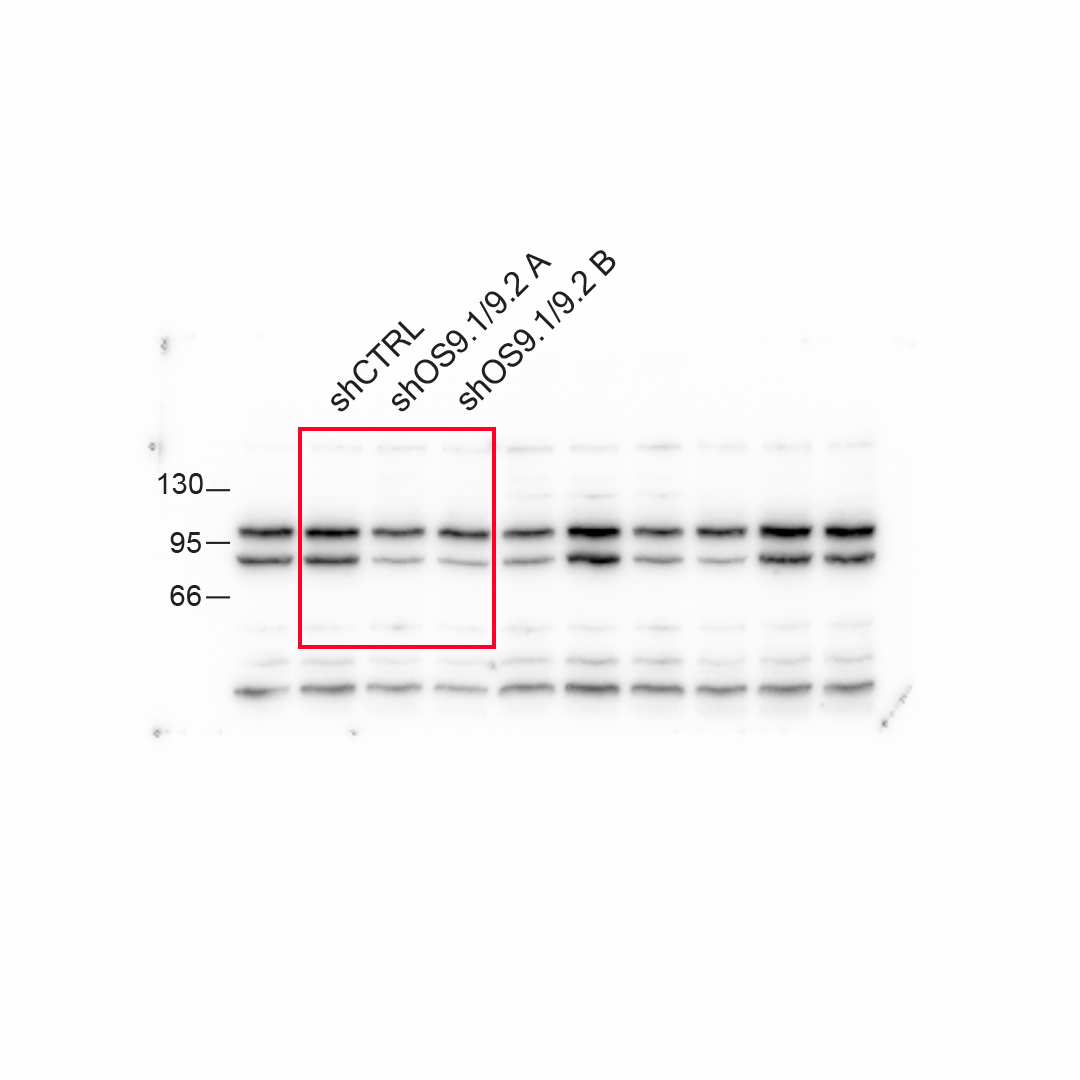

Supplement: Supplementary file 2 — Source data Fig. 3 [file 44319_2024_165_MOESM2_ESM.zip › Figure 3/3D/WB OS9.tif]

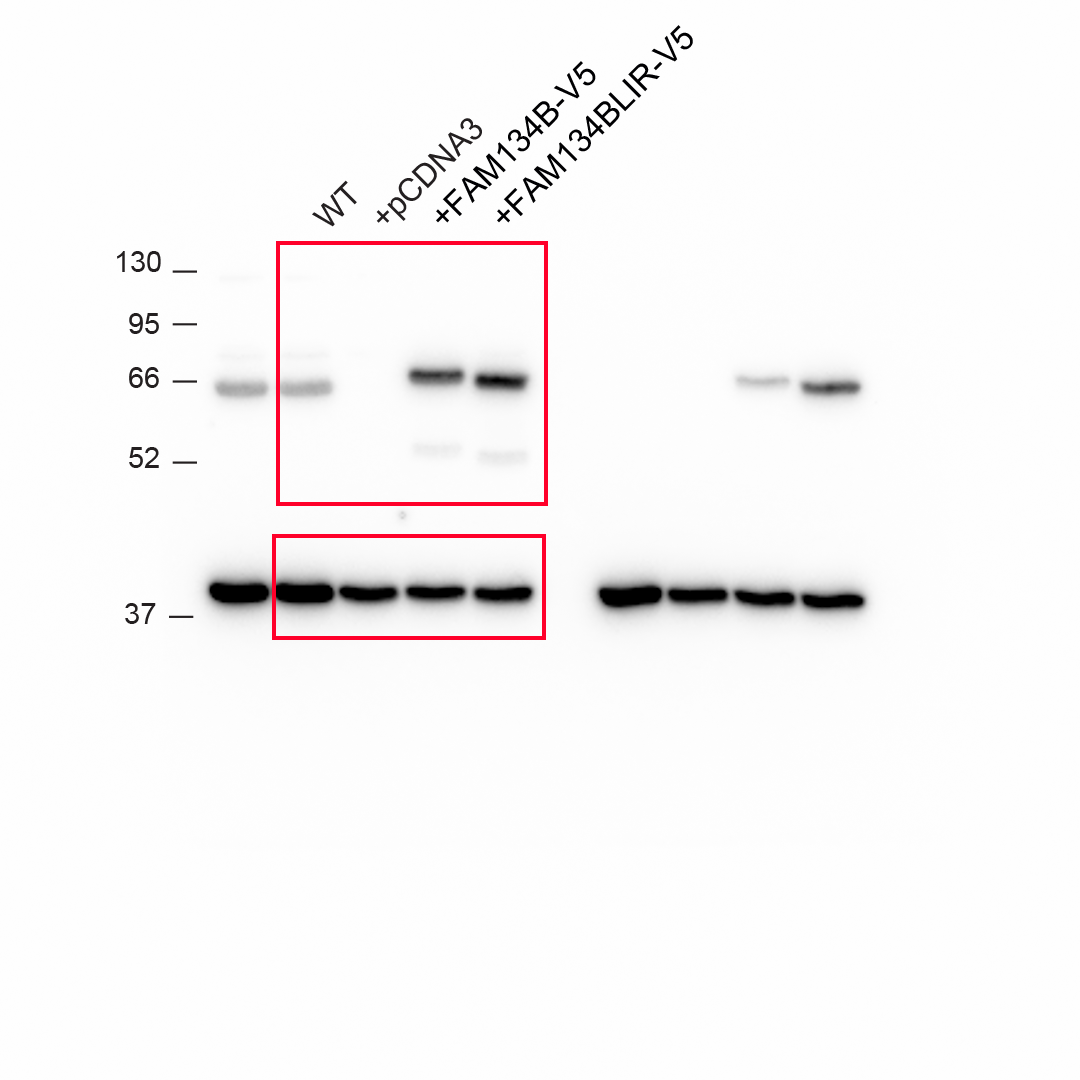

Supplement: Supplementary file 3 — Source data Fig. 4 [file 44319_2024_165_MOESM3_ESM.zip › Figure 4/4A/WB.tif]

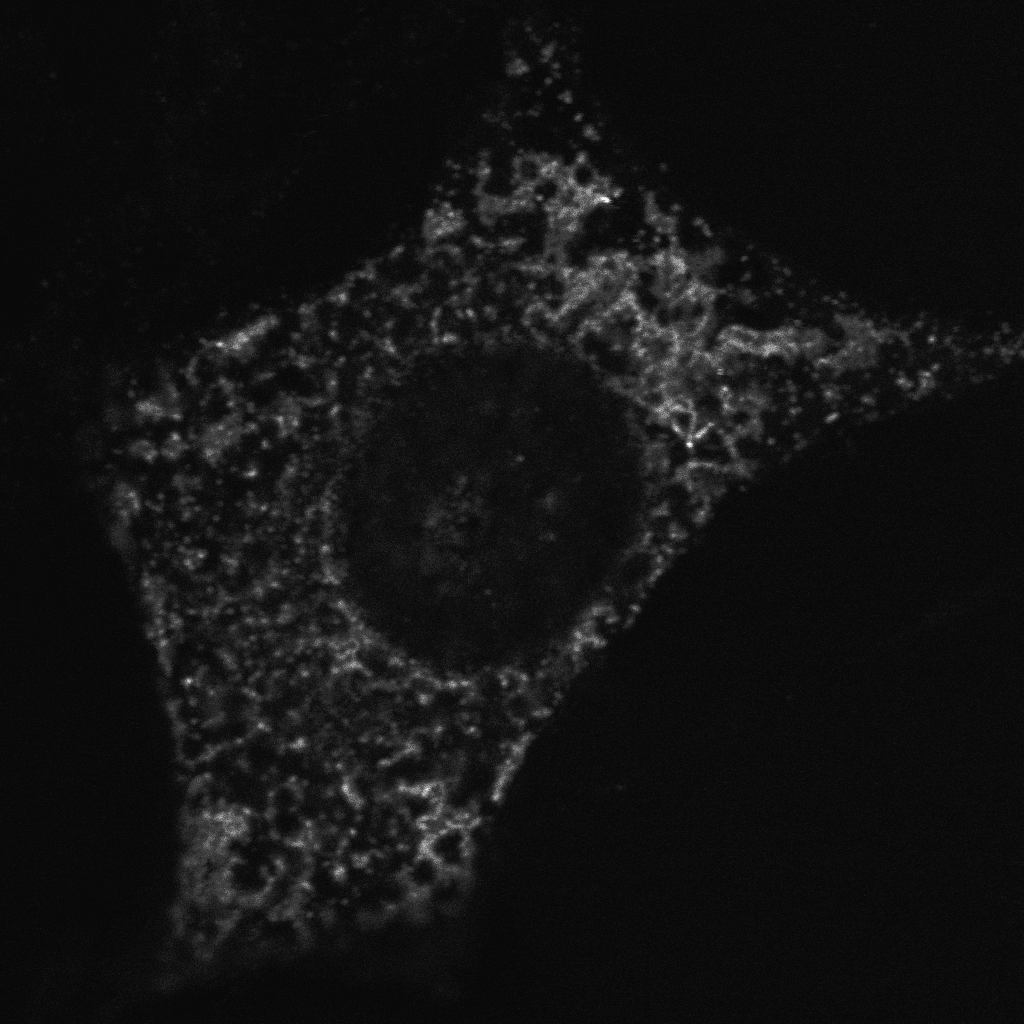

Supplement: Supplementary file 4 — Source data Fig. 5 [file 44319_2024_165_MOESM4_ESM.zip › Figure 5/5A/ha.tif]

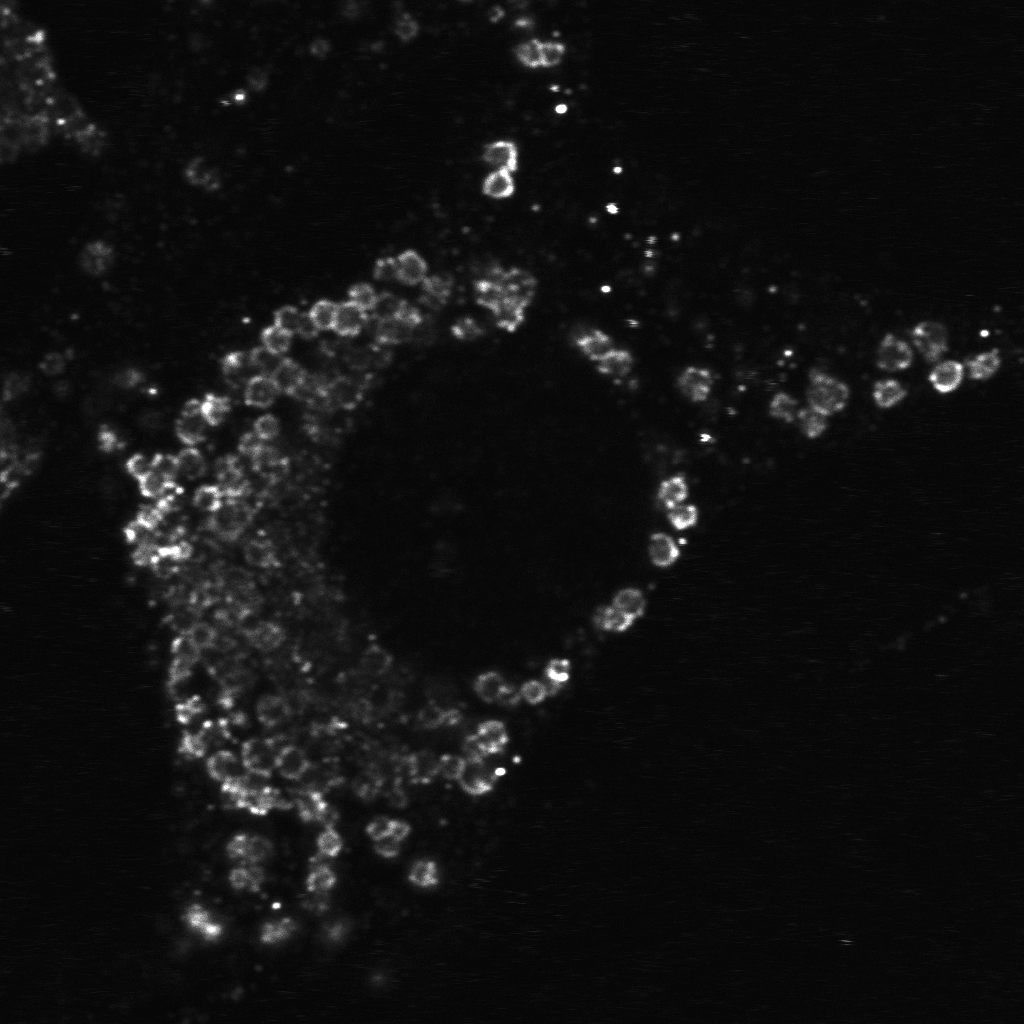

Supplement: Supplementary file 4 — Source data Fig. 5 [file 44319_2024_165_MOESM4_ESM.zip › Figure 5/5A/lamp .tif]

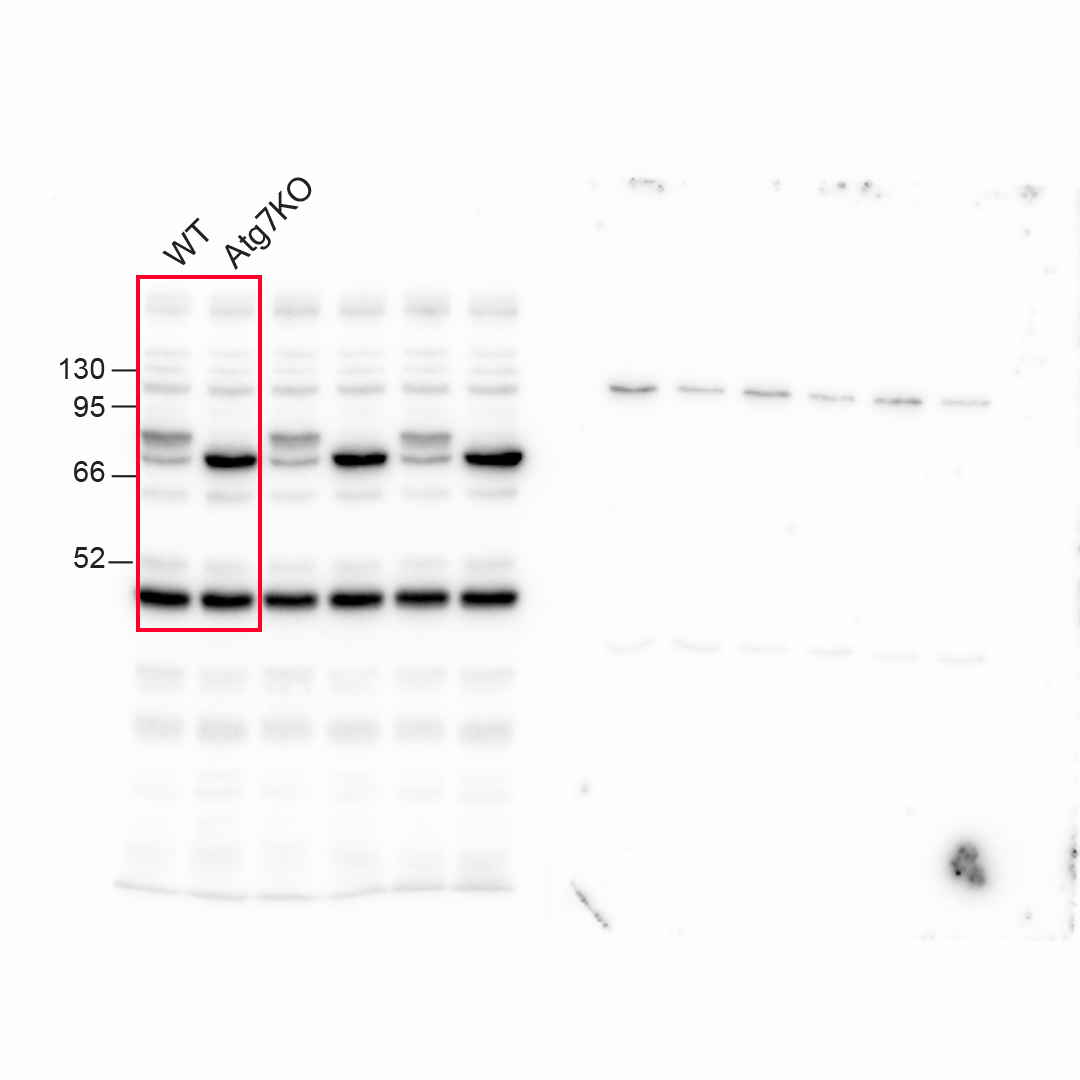

Supplement: Supplementary file 4 — Source data Fig. 5 [file 44319_2024_165_MOESM4_ESM.zip › Figure 5/5D/WB ATG7.tif]

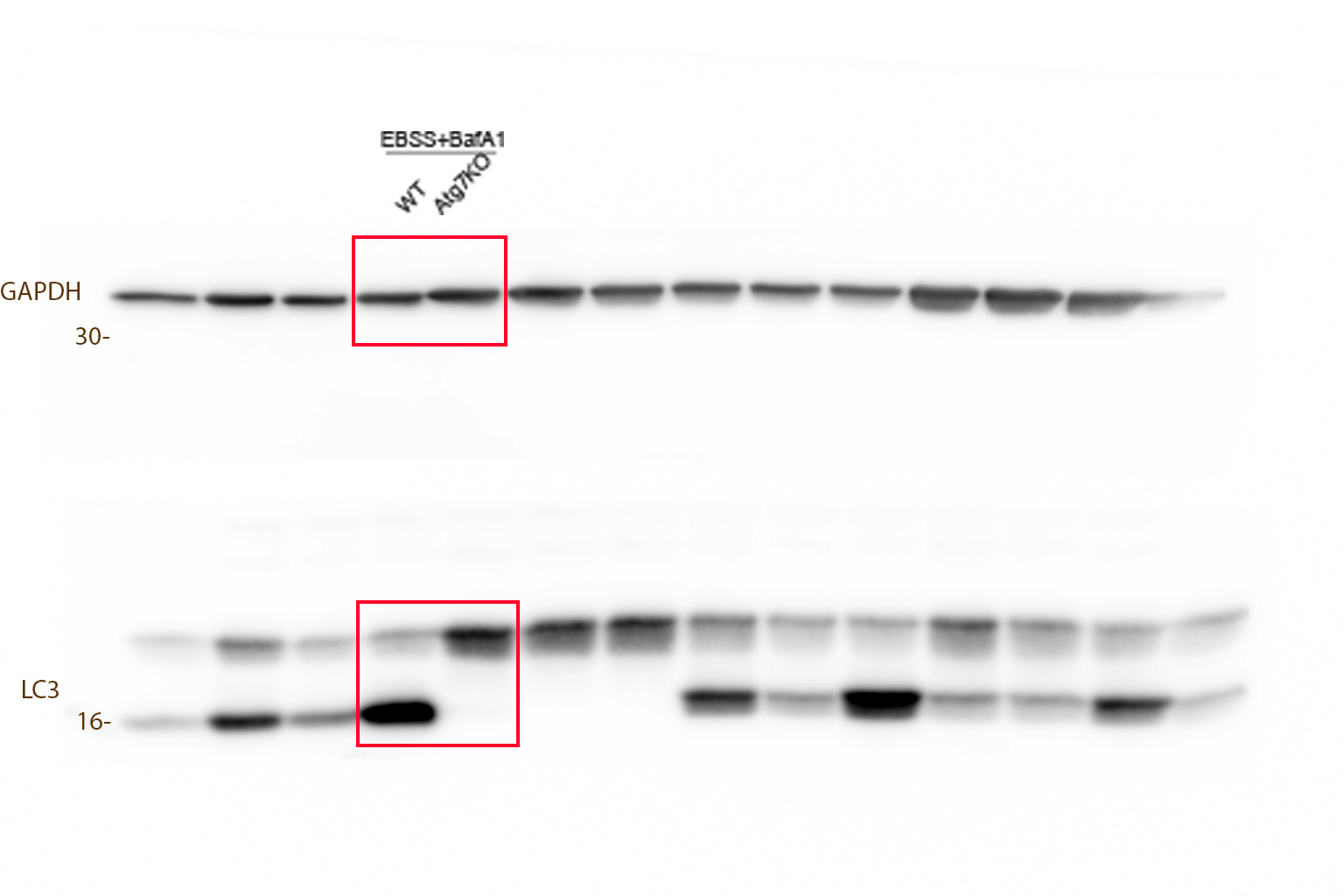

Supplement: Supplementary file 4 — Source data Fig. 5 [file 44319_2024_165_MOESM4_ESM.zip › Figure 5/5D/WB LC3.tif]

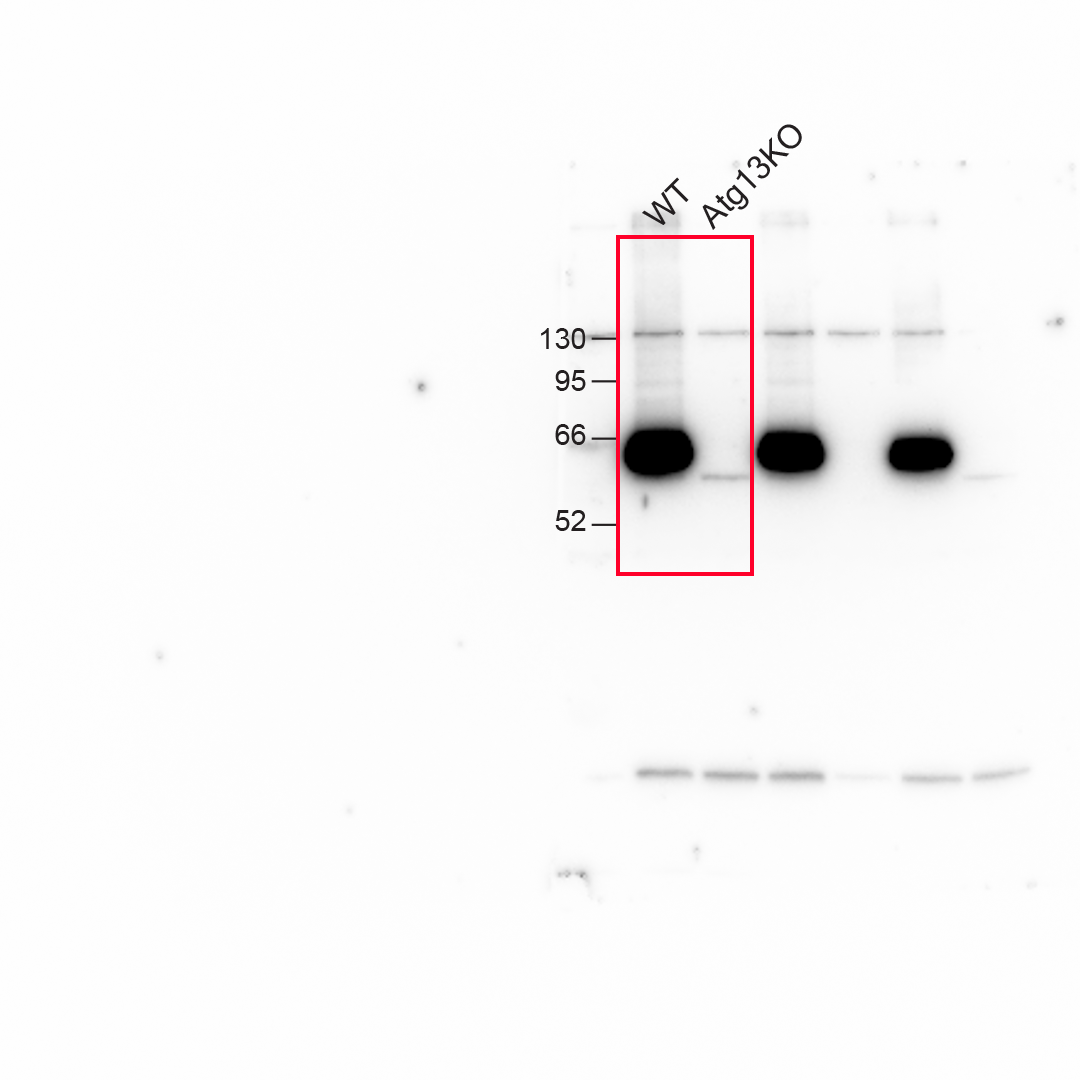

Supplement: Supplementary file 4 — Source data Fig. 5 [file 44319_2024_165_MOESM4_ESM.zip › Figure 5/5F/WB.tif]

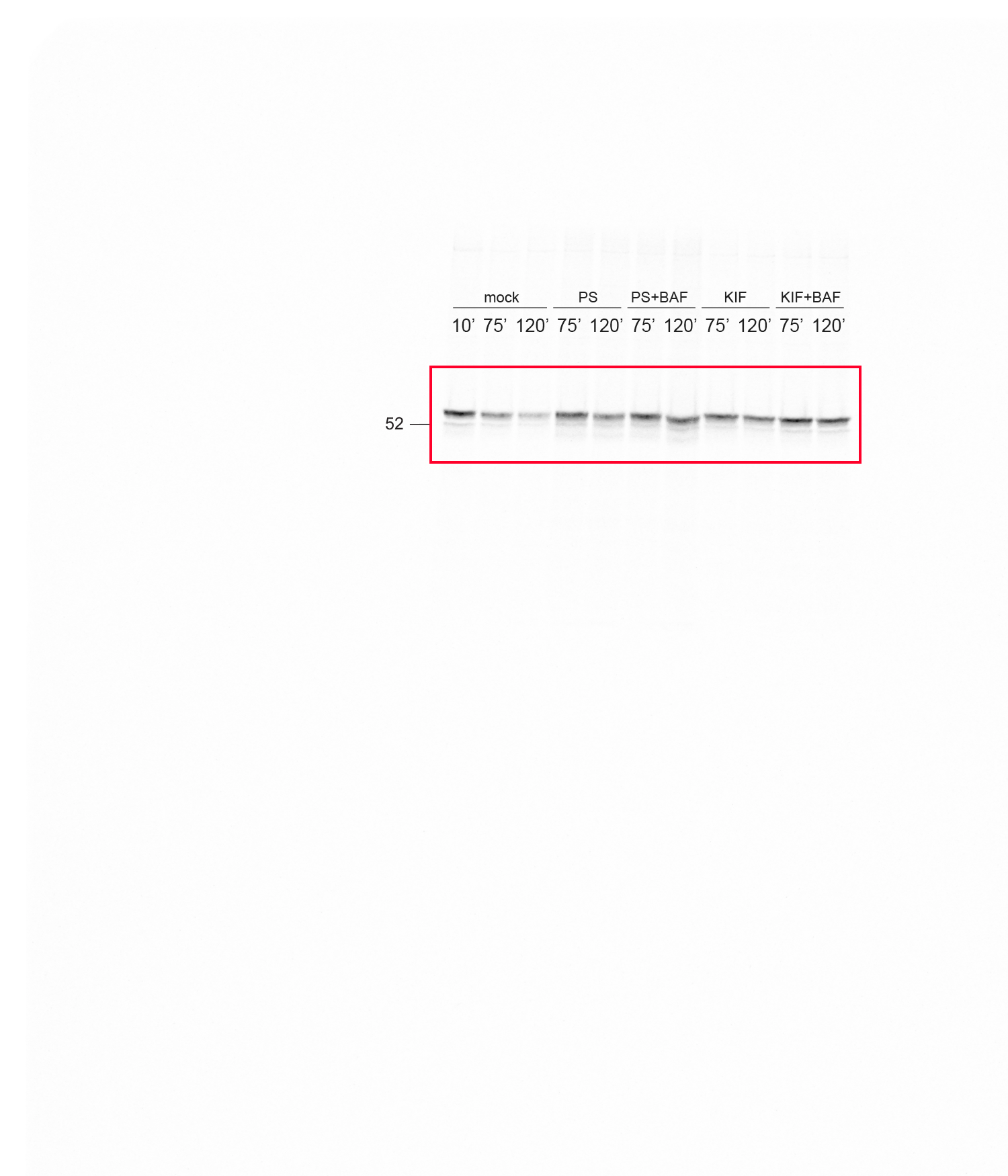

Supplement: Supplementary file 5 — Source data Fig. 6 [file 44319_2024_165_MOESM5_ESM.zip › Figure 6/6A/gel 35S.tif]
